# Hospital Contracts and Prices

**Published:** 1910-05-28

**Authors:** 


### SPECIAL DEPARTMENTAL ARTICLE.

**Contributions and questions on matters affecting this department of an institution are invited.**

### HOSPITAL CONTRACTS AND PRICES.

### VII.—PRICES OF WARD EQUIPMENT.

WE dealt last week with forms of contract for furniture. We now draw attention to what may be considered fair and reasonable prices for reasonable articles of ward equipment, nothing of an extravagant nature being included. A list has been taken of all articles (excepting linen, bedding, and instruments) in use in a ward of a large hospital, and the prices actually paid for them noted. The number of each article required to stock a ward has not been given, as this depends in great measure upon the number of patients a ward is intended to accommodate:—

| | £ | s. | d. |
|---|---|---|---|
| Bags, ice | 0 | 4 | 0 |
| Baskets, balcony | 0 | 2 | 9 |
| Baskets, bread | 0 | 6 | 0 |
| Baskets, medicine | 0 | 6 | 6 |
| Baskets, paper | 0 | 1 | 11 |
| Baskets, wine | 0 | 4 | 0 |
| Baths, arm | 0 | 8 | 6 |
| Bedrests | 0 | 13 | 6 |
| Bedrests, cords for | 0 | 0 | 10½ |
| Bedsteads and spring mattresses | 1 | 8 | 6 |
| Bedstead carriage | 5 | 15 | 0 |
| Bedstead, self-lifters, movable | 0 | 7 | 0 |
| Bedstead, tent, fittings for | 0 | 9 | 6 |
| Bedstops | 0 | 2 | 10 |
| Benches | 1 | 6 | 0 |
| Bells, hand | 0 | 2 | 0 |
| Blinds and rollers | 0 | 7 | 0 |
| Blocks, bed | 0 | 2 | 6 |
| Boards, bath | 0 | 2 | 7 |
| Boards, black, on stand | 4 | 0 | 0 |
| Boards, bread | 0 | 2 | 3 |
| Boards, chart, etc., aluminium | 0 | 2 | 0 |
| Boards, fracture | 0 | 1 | 3 |
| Boards, plaster | 0 | 4 | 0 |
| Boards, poultice | 0 | 2 | 0 |
| Boards, raw meat | 0 | 2 | 0 |
| Boards, steriliser | 0 | 1 | 0 |
| Boards, tea urn | 0 | 1 | 0 |
| Books, devotional, Bible | 0 | 0 | 6 |
| Bottles, hot-water, indiarubber | 0 | 4 | 6 |
| Boxes, alms | 0 | 9 | 0 |
| Boxes, dressing | 0 | 16 | 0 |
| Boxes, housemaids' | 0 | 3 | 2 |
| Boxes, ice | 2 | 0 | 0 |
| Boxes, knife | 0 | 12 | 6 |
| Boxes, note-case, aluminium | 0 | 4 | 4 |
| Boxes, plaster | 0 | 4 | 0 |
| Boxes, plaster bandage | 0 | 4 | 9 |
| Boxes, salt | 0 | 5 | 6 |
| Boxes, soap and soda | 0 | 10 | 9 |
| Boxes, tow | 0 | 7 | 0 |
| Bowls, blacklead | 0 | 0 | 4½ |
| Cans, beef-tea | 0 | 4 | 6½ |
| Cans, broth, etc. | 0 | 4 | 6 |
| Cans, gravy, etc. | 0 | 2 | 0 |
| Cans, milk | 0 | 6 | 6 |
| Cases, note | 0 | 0 | 10 |
| Cases, stationery | 0 | 8 | 4 |
| Chairs, easy | 2 | 2 | 5 |
| Chairs, elbow (sisters') | 0 | 10 | 6 |
| Chairs, heart | 5 | 5 | 0 |
| Chairs, wheel | 2 | 10 | 6 |
| Chairs, Windsor, ward, etc. | 0 | 2 | 4 |
| Cleaning Articles:— | | | |
| Brooms, bass | 0 | 0 | 11 |
| Brushes, long hair sweeping, large | 0 | 5 | 10 |
| Cleaning Articles (*cont.*):— | | | |
| Brushes, long hair sweeping, small | 0 | 3 | 1 |
| Brushes, bed | 0 | 0 | 8½ |
| Brushes, blacklead | 0 | 0 | 1½ |
| Brushes, bottle | 0 | 0 | 3¼ |
| Brushes, coil | 0 | 0 | 11 |
| Brushes, clothes | 0 | 3 | 0 |
| Brushes, dust | 0 | 0 | 9 |
| Brushes, feather | 0 | 0 | 4 |
| Brushes, hair | 0 | 3 | 6 |
| Brushes, hearth | 0 | 1 | 0½ |
| Brushes, lavatory, bristles | 0 | 0 | 7 |
| Brushes, nail | 0 | 0 | 1½ |
| Brushes, rubbers, floor | 0 | 16 | 6 |
| Brushes, saucepan | 0 | 0 | 2¼ |
| Brushes, scrubbing, large | 0 | 0 | 5¼ |
| Brushes, scrubbing, small | 0 | 0 | 5 |
| Brushes, stove | 0 | 0 | 9¾ |
| Brushes, Turk's head | 0 | 2 | 7 |
| Combs, long | 0 | 1 | 0 |
| Combs, small | 0 | 0 | 5 |
| Mops, wool | 0 | 0 | 2½ |
| Pails, galvanised | 0 | 1 | 3 |
| Pans, dust | 0 | 1 | 2 |
| Squeegees | 0 | 3 | 2 |
| Colanders | 0 | 0 | 10½ |
| Corks, bath | 0 | 3 | 6 |
| Cots | 1 | 16 | 0 |
| Couches | 4 | 0 | 0 |
| Cradles, bed, body | 0 | 5 | 9 |
| Cradles, leg | 0 | 5 | 6 |
| Crockeryware, enamelled iron, and glass:— | | | |
| Basins, hand, E.I., doctors' | 0 | 4 | 6 |
| Basins, hand, E.I. patients' | 0 | 1 | 1¾ |
| Basins, soup, crockery | 0 | 0 | 2 |
| Bins, bread, E.I. | 0 | 6 | 3 |
| Bins, soiled linen, E.I. | 2 | 14 | 0 |
| Bottles, hot-water, earthenware | 0 | 1 | 6 |
| Bottles, water, and glass | 0 | 1 | 1 |
| Bowls, dressing | 0 | 0 | 10 |
| Bowls, dressing, kidney, crockery | 0 | 1 | 3 |
| Bowls, dressing, kidney, glass | 0 | 0 | 9 |
| Bowls, dressing, soiled, E.I. | 0 | 2 | 1¾ |
| Bowls, glass, amber or white | 0 | 0 | 8 |
| Bowls, lotion, E.I. | 0 | 0 | 7½ |
| Bowls, lotion, E.I., lipped crockery | 0 | 0 | 3½ |
| Bowls, vomit, lipped crockery | 0 | 0 | 7 |
| Cups, egg | 0 | 0 | 2 |
| Dishes, dressing, large lipped | 0 | 3 | 6 |
| Dishes, soap, E.I., doctors' | 0 | 0 | 9 |
| Dishes, soap, E.I., patients' | 0 | 0 | 6¼ |
| Ewers, E.I. | 0 | 2 | 0 |
| Expectorators, crockery | 0 | 0 | 5¼ |
| Feeders, crockery | 0 | 0 | 6½ |
| Funnels, glass | 0 | 0 | 3 |
| Glasses, medicine | 0 | 0 | 3½ |
| Glasses, minim | 0 | 0 | 2½ |
| Glasses, specimen, numbered, lettered for each bed | 0 | 0 | 7 |
| Glasses, specimen, large | 0 | 1 | 0 |
| Inhalers | 0 | 0 | 7½ |
| Irrigators, crockery | 0 | 3 | 0 |
| Irrigators, enamelled iron | 0 | 3 | 3 |
| Irrigators, glass | 0 | 3 | 0 |
| Jars, butter, crockery | 0 | 1 | 9 |
| Jars, glass | 0 | 1 | 4 |
| Jars, ointment, etc. | 0 | 2 | 8 |
| Jugs, gallon milk, crockery | 0 | 1 | 8 |
| Jugs, ½-gallon lotion, crockery | 0 | 1 | 2 |
| Jugs, 8-pint Parisian, E.I. | 0 | 2 | 0 |
| Jugs, pint, graduated, crockery | 0 | 2 | 9 |
| Measures, glass, 20-oz. | 0 | 0 | 8 |

| Crockeryware, &c. (*cont.*) :— | £ | s. | d. |
|---|---|---|---|
| Mugs, pint ... | 0 | 0 | 2¼ |
| Mugs, half-pint | 0 | 0 | 2 |
| Mugs, nurses' | 0 | 0 | 3 |
| Pails, refuse, E.I. | 0 | 3 | 6 |
| Pails, slop, straight, with valve cover, E.I. ... | 0 | 4 | 9 |
| Pails, soiled dressing, E.I. | 0 | 6 | 6 |
| Pails, test room, E.I. | 0 | 3 | 0 |
| Pans, bed, E.I. | 0 | 4 | 9 |
| Pans, bed, slipper, crockery | 0 | 2 | 4 |
| Plates, dinner | 0 | 0 | 2½ |
| Plates, tea ... | 0 | 0 | 1½ |
| Slabs, marble, for ointment | 0 | 4 | 6 |
| Syringe, glass | 0 | 0 | 3½ |
| Urinals | 0 | 1 | 0 |
| Urinals, boats | 0 | 1 | 3 |
| Cruet, fittings, mustard | 0 | 2 | 8 |
| Cruet, fittings, pepper | 0 | 1 | 4 |
| Cruet, fittings, salt ... | 0 | 0 | 2½ |
| Crutchpads | 0 | 2 | 6 |
| Cupboards, poison, etc. | 2 | 10 | 0 |
| Dredgers | 0 | 2 | 8 |
| Dressers, kitchen | 5 | 0 | 0 |
| Extensions, complete with weights | 0 | 10 | 0 |
| Fire apparatus, Minimax ... | 2 | 4 | 0 |
| Forks, dinner | 0 | 0 | 3 |
| Forks, toasting | 0 | 0 | 4 |
| Funnels, tin ... | 0 | 0 | 7 |
| Guards, fire ... | 0 | 4 | 6 |
| Holders for toilet rolls | 0 | 0 | 11 |
| Irons, fire, pokers | 0 | 0 | 11 |
| Irons, fire, shovels | 0 | 0 | 5 |
| Irons, fire, tongs | 0 | 0 | 7½ |
| Irrigators (constant), metal (special), and stand | 3 | 10 | 0 |
| Kettles | 0 | 5 | 0 |
| Knives, dinner | 0 | 0 | 3¼ |
| Ladles ... | 0 | 0 | 6 |
| Lamps, electric, bell ... | 1 | 0 | 0 |
| Lamps, electric, McDonald | 2 | 0 | 0 |
| Lamps, stand ... | 2 | 7 | 0 |
| Lamps, table ... | 1 | 15 | 0 |
| Lockers ... | 1 | 15 | 0 |
| Machine, bandage winding ... | 0 | 6 | 6 |
| Machine, bandage winding (donkey) | 1 | 6 | 6 |
| Machine, bread cutting | 1 | 5 | 0 |
| Machine, weighing ... | 10 | 0 | 0 |
| Mats, asbestos | 0 | 0 | 1½ |
| Mats, door, rubber ... | 0 | 5 | 9 |
| Mats, kneeling | 0 | 0 | 10½ |
| Measures, milk, ½-pint | 0 | 0 | 8 |
| Microscopes | 7 | 0 | 0 |
| Mirrors ... | 0 | 8 | 0 |
| Pans, frying ... | 0 | 1 | 0 |
| Pans, sauce, iron | 0 | 2 | 4½ |
| Pegs, hat and coat | 0 | 0 | 6 |
| Picks, ice | 0 | 1 | 2 |
| Poles, window | 0 | 2 | 0 |
| Racks, plate ... | 1 | 5 | 0 |
| Racks, clothes, galvanised iron (patients') | 7 | 10 | 0 |
| Rails, mackintosh, sets | 1 | 10 | 0 |
| Receptacles, ash | 0 | 12 | 6 |
| Rollers, towel, and brackets | 0 | 2 | 3 |
| Screens, four-fold | 1 | 11 | 3 |
| Scuttles, coal ... | 0 | 2 | 0 |
| Seats, balcony | 0 | 16 | 0 |
| Slices, fish | 0 | 0 | 9 |
| Spatulæ, poultice | 0 | 1 | 6 |
| Spatulæ, tongue | 0 | 2 | 8 |
| Spoons, dessert | 0 | 0 | 5¼ |
| Spoons, table ... | 0 | 0 | 5½ |
| Spoons, tea | 0 | 0 | 2½ |
| Stands, ink | 0 | 2 | 3 |
| Stands, oxygen | 0 | 15 | 0 |
| Stands, specimen glass | 0 | 10 | 6 |
| Stands, test ... | 0 | 17 | 9 |
| Steps, lattice ... | 0 | 5 | 11 |
| Sterilisers, electric ... | 10 | 0 | 0 |
| Syringes (Higginson's) | 0 | 1 | 6 |
| Tables, bed ... | 0 | 9 | 3 |
| Tables, glass and iron, on wheels ... | 3 | 17 | 0 |
| Tables, kitchen | 1 | 7 | 3 |
| Tables, sisters' | 1 | 10 | 0 |
| Tables, ward ... | 2 | 0 | 0 |
| Teapots | 0 | 0 | 10 |
| Thermometer, bath ... | 0 | 0 | 7½ |
| Thermometer, clinical | 0 | 0 | 5½ |
| Thermometer, wall ... | 0 | 1 | 3 |
| Tins, dinner, jacketed | 1 | 10 | 0 |
| Tins, dinner, cold-meat | 0 | 3 | 6 |
| Tins, arrowroot | 0 | 0 | 9 |
| Tins, dressing, sterilised | 0 | 15 | 0 |
| Tins, mustard ... | 0 | 0 | 5¾ |
| Tins, pepper ... | 0 | 0 | 5¾ |
| Tins, sugar | 0 | 5 | 0 |
| Tins, tea | 0 | 2 | 0 |
| Trays, brass ... | 0 | 8 | 6 |
| Trays, metal ... | 0 | 2 | 0 |
| Trays, zinc, for glass and iron table | 0 | 2 | 0 |
| Trolleys, ambulance ... | 7 | 5 | 0 |
| Tubs, wash-up | 0 | 3 | 10 |
| Urns, tea, copper | 2 | 0 | 0 |
| Wagons, book ... | 2 | 16 | 6 |
| Wagons, coal ... | 5 | 10 | 0 |
| Wagons, dinner | 3 | 16 | 6 |
| Wagons, dressing | 9 | 0 | 0 |
| Wagons, washstand (complete with 1 lotion receptacle, 2 bowls, 2 Parisian jugs, 2 pails) ... | 12 | 0 | 0 |

With regard to articles purchased under the sub-head "Hardware, Crockery, Brushes, &c." we find that there is no uniform system of purchasing. Hospitals have adopted principles which they have found to be most convenient. Where store-room accommodation is sufficient, crockeryware is frequently purchased once a year, competitive prices being obtained. In other institutions with less storage room a contract exists extending over three or five years, goods being ordered as required.

The same systems are in vogue with respect to brushes and certain articles of hardware, and there is no doubt that with either of these methods of purchasing, hospitals obtain reasonable prices.

